# Temperature and pressure limits of guanosine monophosphate self-assemblies

**DOI:** 10.1038/s41598-017-10689-0

**Published:** 2017-08-29

**Authors:** Mimi Gao, Balasubramanian Harish, Melanie Berghaus, Rana Seymen, Loana Arns, Scott A. McCallum, Catherine A. Royer, Roland Winter

**Affiliations:** 1 Physical Chemistry I - Biophysical Chemistry, Faculty of Chemistry and Chemical Biology, Technical University Dortmund, Otto-Hahn-Street 4a, 44227 Dortmund, Germany; 2 Center for Biotechnology & Interdisciplinary Studies, Rensselaer Polytechnic Institute, Troy, New York 12180 United States; 3 NMR Facility Center for Biotechnology and Interdisciplinary Science, Rensselaer Polytechnic Institute, Troy, NY USA

## Abstract

Guanosine monophosphate, among the nucleotides, has the unique property to self-associate and form nanoscale cylinders consisting of hydrogen-bonded G-quartet disks, which are stacked on top of one another. Such self-assemblies describe not only the basic structural motif of G-quadruplexes formed by, e.g., telomeric DNA sequences, but are also interesting targets for supramolecular chemistry and nanotechnology. The G-quartet stacks serve as an excellent model to understand the fundamentals of their molecular self-association and to unveil their application spectrum. However, the thermodynamic stability of such self-assemblies over an extended temperature and pressure range is largely unexplored. Here, we report a combined FTIR and NMR study on the temperature and pressure stability of G-quartet stacks formed by disodium guanosine 5′-monophosphate (Na_2_5′-GMP). We found that under abyssal conditions, where temperatures as low as 5 °C and pressures up to 1 kbar are reached, the self-association of Na_2_5′-GMP is most favoured. Beyond those conditions, the G-quartet stacks dissociate laterally into monomer stacks without significantly changing the longitudinal dimension. Among the tested alkali cations, K^+^ is the most efficient one to elevate the temperature as well as the pressure limits of GMP self-assembly.

## Introduction

The discovery in 1953 of the right-handed double helical structure and the Watson-Crick base pairs of deoxyribonucleic acid (DNA) gave birth to modern molecular biology^1–3^. However, in recent years it has become evident that DNA can also adopt a variety of non-canonical conformations *in vivo* including G-quadruplex structures^4^, which are thought to be functionally crucial for genome integrity and stability. G-quadruplex structures have emerged to be substantial nucleic acid based control and sensing elements involved in regulating and altering transcription, replication, translation and genome stability^5–9^. Targeting G-quadruplexes in gene promotors has also drawn attention in anticancer strategies^7, 10^. Potential G-quadruplex sequences are highly conserved and have been demonstrated to exist throughout the functional parts of prokaryotic and eukaryotic genomes suggesting importance in biological evolution^5, 7^. They are formed in G-rich regions of DNA or RNA sequences. However, there are fundamental differences between RNA and DNA G-quadruplexes. The additional 2′-hydroxyl-group in the ribose sugar causes greater hydration and enhanced stability of the RNA variant^6, 11^. However, a very recent study pointed out that the formation of RNA G-quadruplexes is overwhelmingly prevented in eukaryotic cells, whereas the same sequences ectopically expressed in *Escherichia coli* fold into the G-quadruplex conformation, suggesting the presence of a very effective molecular machinery in eukaryotes to maintain such structures in the unfolded state^12^. Similarly, the formation of DNA G-quadruplexes in heterochromatin is also suppressed, whereas G-quadruplexes can be found in nucleosome-depleted regions and their formation is associated with increased transcriptional activity^13^. The structural topology of G-quadruplexes is highly polymorphic and depends on the number, orientation and loop structure of the G-rich chains during G-quadruplex folding^14^. Despite their structural polymorphism, they share the common feature of forming cyclic, planar and hydrogen-bonded guanine tetramers known as the G-quartets which are stacked on top of one another forming a right-handed four-stranded helical structure. In the helix, adjacent G-quartets are twisted by 30° and separated by 3.4 Å^15–17^. The G-quadruplex structure is stabilized by monovalent cations which are sandwiched between the G-quartets in the central cavity coordinating the eight guanine O6 atoms^18–23^. However, in case of the smallest alkali cation Na^+^, it can exist in- or out-of-plane and move in and out of the octamer up to 108 times per second^20, 23–25^. Those cation-dipole interactions stabilize the self-complementary hydrogen bonds of guanine and the base stacking interactions along the helix. The cation not only defines the stability of G-quadruplex structures, but also plays an important role in modulating their structural polymorphism^26, 27^.

**Figure 1:**
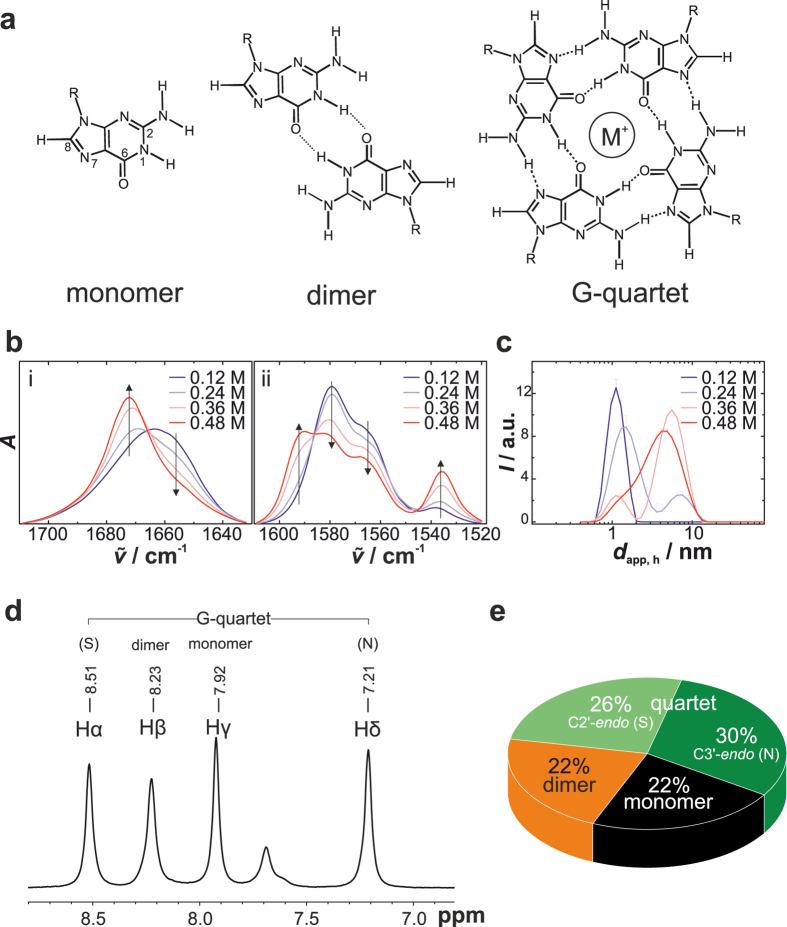
Concentration-dependent self-assembly of disodium 5′-guanosine monophosphate (Na_2_5′-GMP) at pH 8. (**a**) Schematic structure of 5′-GMP monomer, dimer and G-quartets. For clarity, only the chemical structure of the guanine base is shown. The cation M^+^ can be in-plane or out-of-plane of the G-quartet. (**b**) Area normalized FTIR spectra of Na_2_5′-GMP in D_2_O at different concentrations: (i) in the range of 1700–1640 cm^−1^ (C=O stretch vibration), and (ii) in the range of 1600–1520 cm^−1^ at 282 K (C=N and C=C ring vibration). Arrows indicate the absorbance change upon self-assembly. (**c**) DLS diagrams of Na_2_5′-GMP in H_2_O at different concentrations and 282 K. Hydrodynamic diameters calculated based on spherical symmetric particles and using the method of cumulants. Error bars indicate mean ± s.d. of three scans. (**d**) ^1^H NMR spectrum of the H8 region and (**e**) the corresponding population distribution for 0.48 M Na_2_5′-GMP at 278 K.

Interestingly, since the early 1960s, long before the existence of telomeres and their G-quadruplex structures had been shown^17, 28^, the unique ability of guanosine 5′-monophosphate (5′-GMP) to undergo self-association, thereby forming right-handed helices with G-quartets stacking along the helix axis, was already known^15, 25, 29^. The propensity of self-assembly and the size of aggregates depend on the sample concentration, solution acidity and presence of alkali cations^25, 29–32^. At acidic pH, the self-association of 5′-GMP can even lead to the formation of anisotropic gels^33^. A recent NMR study revealed that three types of stacked 5′-GMP aggregates can generally coexist upon self-assembly using monomers, dimers and G-quartets as building blocks (Fig. 1a)^34^. The monomeric and dimeric species undergo exchange on the ms time scale, whereas the tetrameric species are rather kinetically stable^30, 34^. The size of the self-aggregates has been shown to be on the nanometer scale corresponding to tens of quartets per stack^31, 32, 35^. The 5′-GMP quartets are stacked in a head-to-tail manner with alternating C2′-*endo* and C3′-*endo* sugar puckers along the helical strand forming a chiral cylinder^34^. The alternating sugar conformations allow further stabilization of the helix via P-O^−^···H-O_2/3_′ hydrogen bonds along the helical strand which are reminiscent of the phosphodiester bonds in DNA or RNA^34^. Self-assembly of 5′-GMP is not only determined by Hoogsteen-like base pairing, π-π interaction, the hydrophobic effect and thus release of water, but also by cation binding^36^. The cation-templated self-association requires two different cation binding sites, namely the surface and channel site^37, 38^. The adjacent peripheral phosphate groups are separated by 6.7 Å allowing true ionic binding of a sodium cation to bridge and to partially neutralize the electrostatic repulsion of the two doubly negatively charged groups^34, 37^. In contrast, due to steric reasons, potassium and rubidium cations have been shown to undergo only counterion condensation to screen the charge density of the phosphate groups^37^. Consistently, the order of cation binding affinity for the surface site has been found to be Na^+^ > Rb^+^ > K^+^^38^. However, the stability of the G-quartet stacks is predominantly determined by the cation type located in the central channel of the helix. The eight inwardly pointing guanine O6 atoms of two adjacent G-quartets span a volume of ca. 40 Å^3^ serving as the channel binding site which has an affinity order of K^+^ > Rb^+^ > Na^+^^19, 37–40^. *Ab initio* calculations showed that the cation-dipole interaction provides more stabilization than either hydrogen bonding or stacking interaction and diminishes the electrostatic repulsion between the tightly packed cations^41, 42^. For a long time, the cation selectivity of the channel site was explained by the concept of optimal fit^18, 43^. Compared to the smaller Na^+^ and the larger Rb^+^, K^+^ ions were thought to have the optimal size to fit into the central cavity of the G-quartet helix. However, the mechanism of size-selective coordination does not consider the dehydration process of the cation before entering the cavity and the associated free energy cost which has to be balanced by the free energy of the cation-guanine interaction^44–46^. NMR studies and *ab initio* calculations have revealed that the free energy of the cation-guanine interaction follows the trend in charge density of the alkali cations, but the preferred coordination of K^+^ over Na^+^ originates from their relative free energies of hydration^38, 45, 46^. Therefore, the cation affinity for the channel site is controlled by the balance of the free energy cost and gain of cation dehydration and binding, respectively.

Due to its simplicity, the non-covalent self-association of 5′-GMP has drawn unabated attention since its discovery and has become an essential model in many areas including structural biology, medicinal chemistry, supramolecular chemistry and nanotechnology^25^. The G-quartet structures show promise as a scaffold for synthetic ion channels, but also as components in nanotechnology including nanowires, nanomachines, biosensors, and molecular electronic devices^25^. Moreover, in origin-of-life studies, particularly in those where a protein-free RNA world scenario is assumed^47, 48^, the self-assembly of GMP could have served as a platform for prebiotic coalescence of nucleotides^37, 49, 50^. Interestingly, today densely packed guanine crystals can be found in the retinal pigment epithelium of deep-sea fishes focusing light to the photoreceptors^51^. As a popular model for numerous disciplines, the thermodynamic stability of GMP self-assemblies is still largely unexplored, however. Here, we present a comprehensive study to reveal the temperature- and pressure-dependent stability of G-quartet stacks formed by 5′-GMP in the presence of the alkali cations Na^+^, K^+^ and Rb^+^. Using a series of scattering and spectroscopic methods, we show that under abyssal conditions, where temperatures as low as 5 °C and pressures up to 1 kbar are reached, the self-association of Na_2_5′-GMP is most favoured. Beyond those conditions, the G-quartet stacks dissociate laterally into monomer stacks without changing the longitudinal dimension. Hydration, stacking interaction and packing defects play important roles in determining the stability of such supramolecular structures. In the presence of the alkali cations K^+^ and Rb^+^, which bind and replace the Na^+^ cation from the channel cavity of the G-quartet stacks, the temperature and pressure stability of the 5′-GMP quartets is significantly increased.

## Results and Discussions

### Concentration-dependent self-assembly

In the planar G-quartets, hydrogen bonds are formed between N1-H and N2-H as H-donors and O6 and N7 as H-acceptors, whereas dimer formation is based on hydrogen bonds between N1 and O6 (Fig. 1a). Hence, significant shifts and absorbance changes of infrared (IR) bands in the region of 1700–1500 cm^−1^ characteristic of the guanine base can be observed upon 5′-GMP self-assembly^52^. Figure 1b displays concentration-dependent Fourier-transform infrared (FTIR) spectra of Na_2_5′-GMP at pH 8. The band at ${\tilde{v}}_{max}$ = 1657 cm^−1^ has been assigned to the C6=O6 stretch vibration of guanine. Upon 5′-GMP self-association, the carbonyl band is blue shifted to ${\tilde{v}}_{max}$ = 1673 cm^−1^ and becomes sharper. Simultaneously, the ring vibrations of guanine including C=C and C=N stretch vibration are also affected by base pairing and stacking of 5′-GMP, i.e., an intensity increase of the bands at ${\tilde{v}}_{max}$ = 1592 cm^−1^ and 1537 cm^−1^ as well as an intensity decrease of the bands at ${\tilde{v}}_{max}$ = 1582 cm^−1^ and 1568 cm^−1^. Further, we followed the self-assembly of Na_2_5′-GMP via dynamic light scattering (DLS) and obtained qualitative information about the size distribution of 5′-GMP aggregates (Fig. 1c). The cylinder diameter of monomeric and tetrameric 5′-GMP aggregates are approximately 1 and 2.6 nm, respectively^32^. The length of the cylinder is defined by (*n* − 1) × 0.34 nm, where *n* is the number of quartets in the stack. At a concentration of 0.12 M and pH 8, our FTIR and DLS data indicate that Na_2_5′-GMP does not form stacks of dimers or quartets, but non-hydrogen-bonded aggregates, thus very likely monomeric stacks are present only. These findings are in agreement with literature data^32^. Starting from 0.24 M, initial formation of larger hydrogen-bonded 5′-GMP assembled species can be observed. They coexist with the monomeric stacks and their presence increases with the concentration of Na_2_5′-GMP (Fig. 1a and c). To further characterize those assemblies, we applied ^1^H nuclear magnetic resonance (NMR) spectroscopy. Upon self-association of 5′-GMP, ring-current and electrostatic effects induce upfield and downfield shifts of the H8 signal^30^. The four peaks of the H8 region located between 7.1 and 8.6 ppm of the ^1^H NMR spectrum have been assigned to monomeric, dimeric and tetrameric species of 5′-GMP (Fig. 1d)^34, 53^. The “outer” peaks at 8.5 and 7.2 ppm (H_α_ and H_δ_) with equal intensities are attributed to tetrameric 5′-GMP with either a C2′-*endo* or a C3′-*endo* sugar pucker conformation. The H_γ_ peak at 7.9 ppm indicates monomeric 5′-GMP, whereas the H_β_ peak at 8.2 ppm corresponds to dimeric 5′-GMP. By integrating the H8 signals, we could quantify the population distribution for the self-assembly of Na_2_5′-GMP (Fig. 1e). At a concentration of 0.48 M, more than 50% of the 5′-GMP molecules participate in forming G-quartet stacks. Only about 22% of the nucleotides are in the short-lived dimeric state^34^. Previous NMR diffusion experiments have revealed that the number of stacks increases with the nucleotide concentration^32^. Thereby, Wong *et al*. found the mechanism of stacking to be the same for monomers and G-quartets^32^. Hence, the channel cation in G-quartet stacks might be less needed for stacking, but rather to neutralize the electrostatic repulsion of the O6 atoms of the G-quartets. Furthermore, we found the concentration dependence of the Na_2_5′-GMP self-assembly to be affected by temperature (Suppl. Fig. S1), in good agreement with literature data^32^. While initial hydrogen-bonded self-assembly is observable at 0.24 M Na_2_5′-GMP and 282 K, the critical concentration is increased to 0.48 M at 296 K, indicating high entropic costs for the self-association.

### Thermal Stability of 5′-GMP self-assemblies

**Figure 2:**
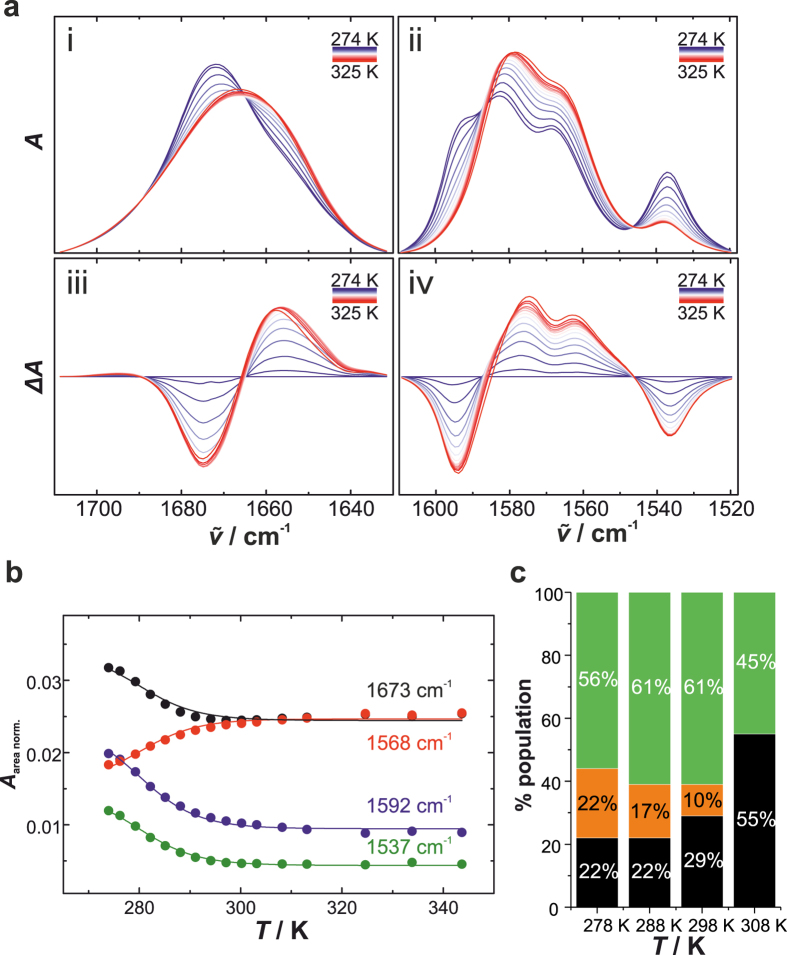
Temperature dependence of 5′-GMP self-assembly. (**a**) Area normalized FTIR spectra of 0.48 M Na_2_5′-GMP in D_2_O as a function of temperature for (i) the C=O stretch vibration, and (ii) the ring vibrations (C=C, C=N), with (iii-iv) the corresponding difference spectra. The absorption spectrum at 274 K was subtracted for the spectra at high temperatures. (**b**) Absorbance intensities at the given wavenumbers as a function of temperature. The lines indicate a global Boltzmann fit to the experimental data. (**c**) Population distribution of 0.48 M Na_2_5′-GMP at different temperatures obtained from ^1^H NMR. Green: G-quartet, orange: dimer, black: monomer.

Previous NMR diffusion experiments have demonstrated that the stacking behaviour of G-quartets and thus the length of the stacks are not modulated by temperature in the range between 278 and 298 K^32^. To yield information about the thermal stability of the hydrogen bonds formed in dimers and G-quartets of 5′-GMP, we performed temperature-dependent FTIR measurements for 0.48 M Na_2_5′-GMP at pH 8 (Fig. 2). IR spectra of both the carbonyl band and the ring vibrations change markedly with increasing temperature, indicating dissociation of the dimers and G-quartets (Fig. 2a). Upon temperature increase, the carbonyl band shifts from 1673 to 1657 cm^−1^ and broadens. In the case of the ring vibrations, the band intensities at 1592 and 1537 cm^−1^ decrease at elevated temperatures, whereas the bands at 1582 and 1568 cm^−1^ show increased intensities and slight red shifts. To better describe the “melting” behaviour, we plotted the band intensities at 1673, 1592, 1568 and 1537 cm^−1^ as a function of temperature (Fig. 2b). The “melting” curves follow the same course, indicating that any of those bands can be used to follow the self-association of 5′-GMP. Assuming an apparent thermodynamic equilibrium between all the hydrogen-bonded and the monomer stacks, a global Boltzmann fit of the IR data was performed. We obtained a melting temperature of *T*_m_ = 281 ± 2 K and a standard-state van’t Hoff enthalpy change of ${\rm{\Delta }}{H}_{{\rm{vH}}}^{{\rm{0}}}$ = 124 ± 8 kJ mol^−1^ for the dissociation reaction of Na_2_5′-GMP. In this thermodynamic model, the contribution of 5′-GMP monomers not participating in the self-association reaction, are not considered in the initial plateau values. Using ^1^H NMR, we obtained comparable trends by analyzing the temperature-dependent population distribution of the monomer, dimer and G-quartet species (Fig. 2c). Although *ab initio* calculations have assigned the Watson-Crick faced guanine dimer (GG3^2^) to be the most stable homo base pair^54^, it is less thermally stable compared to the cation-templated G-quartet. Furthermore, our temperature-dependent DLS measurements suggest that temperatures up to 348 K induce only the dissociation of hydrogen bonds and do not affect the stacking behaviour (Suppl. Fig. S2). The hydrodynamic size of the monomer stacks is hardly changed by the temperature. Taken together, our data reveal that the stacking interaction involved in 5′-GMP self-assembly can withstand a wide range of temperatures, whereas the base pairs are only stable at low temperatures. In an entropy-centered view, the favourable stacking interactions at high temperature can be explained by the hydrophobic effect and thus an entropy gain due to water release, whereas the G-quartet formation is accompanied by conformational entropy loss and thus becomes unfavourable with increasing temperature.

### Effect of pressure on 5′-GMP self-assembly

**Figure 3:**
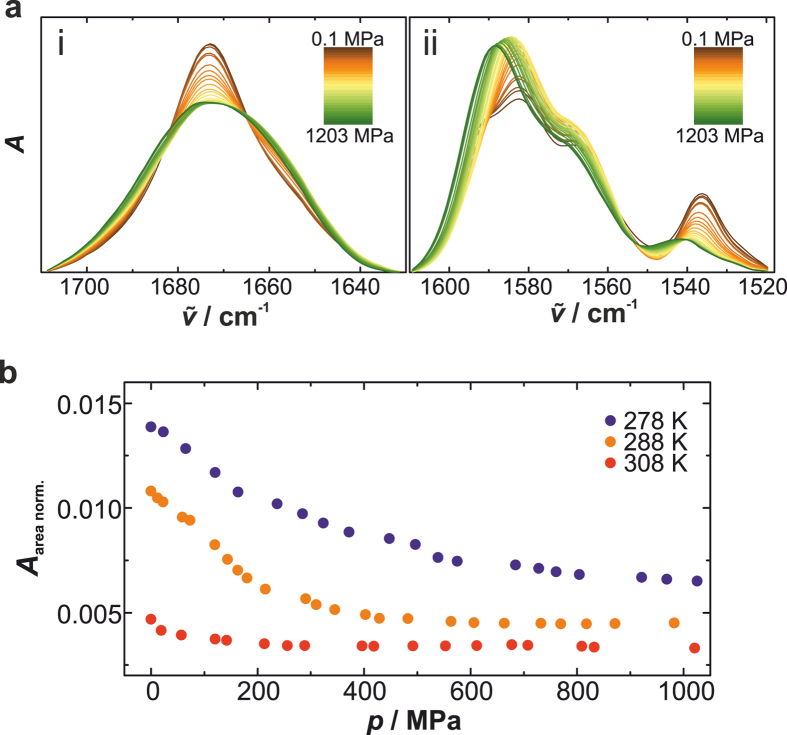
Pressure dependence of 5′-GMP self-assembly. (**a**) Area normalized FTIR spectra of 0.48 M Na_2_5′-GMP in D_2_O and at 288 K as a function of pressure for (i) the C=O stretch vibration, and (ii) the ring vibrations (C=C, C=N). (**b**) Absorbance intensities of the IR band at ~1537 cm^−1^ as a function of pressure upon area normalization.

**Figure 4:**
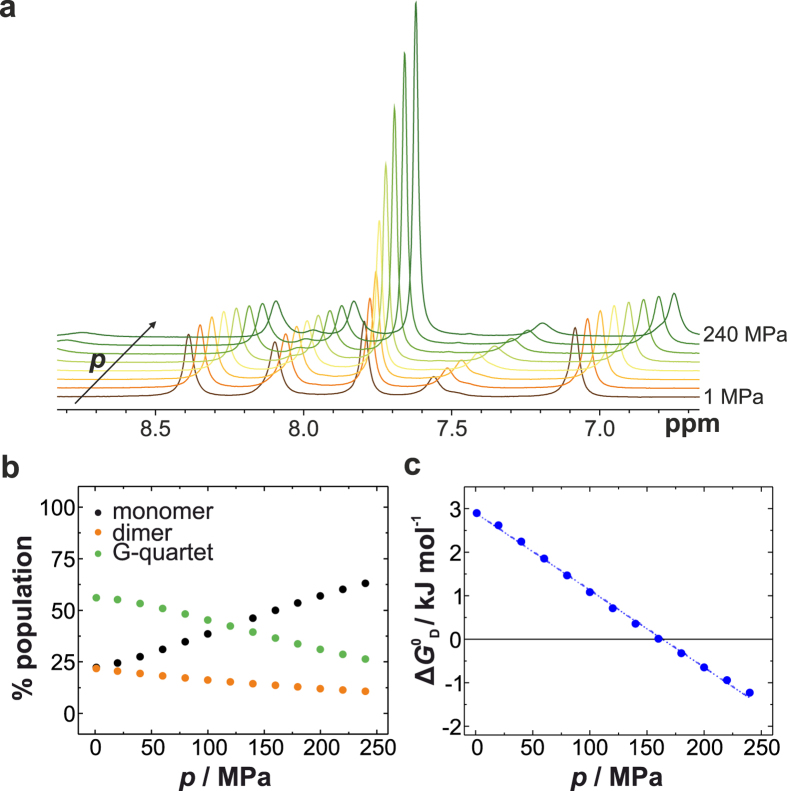
High pressure ^1^H NMR of 5′-GMP self-assembly at 278 K. (**a**) Stack plot of the ^1^H NMR spectra for 0.48 M Na_2_5′-GMP as a function of pressure. (**b**) Pressure-dependent population distribution of 5′-GMP. (**c**) Pressure-dependent standard free energy of the 5′-GMP disassembly. The dashed line is the least-square fit to the data and describes the standard molar volume change of the dissociation process.

**Figure 5:**
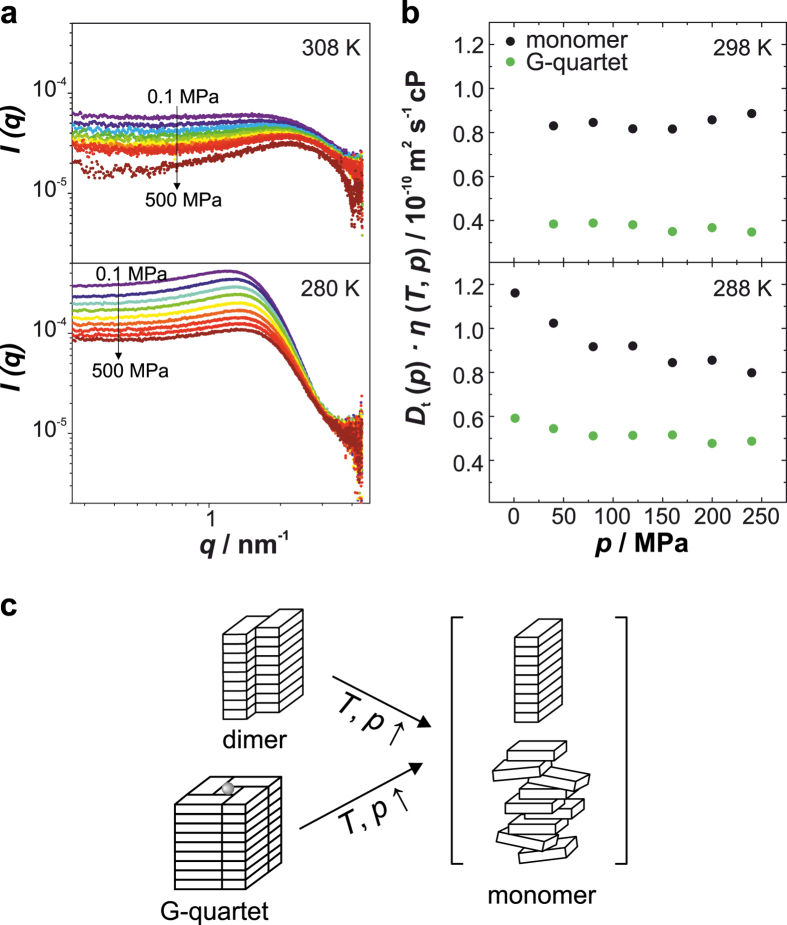
Effect of pressure on the size of 5′-GMP aggregates. (**a**) SAXS intensity profiles of 0.48 M Na_2_5′-GMP in H_2_O as a function of pressure. (**b**) Viscosity corrected translational diffusion coefficients, *D*_t_·*η*, of 5′-GMP monomer and G-quartet stacks as a function of pressure obtained from ^1^H DOSY NMR. (**c**) Tentative illustration of the dissociation process of 5′-GMP self-assemblies induced by temperature and pressure. Of note, the morphology of the monomeric stacks/aggregates is not known.

Pressure acts on molecular systems through changes in specific volume that are due to changes in hydration and packing efficiency^55^. According to *Le Châtelier*’s principle, conformations occupying smaller volumes and processes for which the transition state has a smaller volume than the ground state are favoured under high pressure conditions^56^. In the case of proteins, pressure favours conformations that minimize solvent-excluded void volume and causes water filling of hydrophobic cavities/voids. Furthermore, pressure leads to greater hydration of polar/charged amino acids, resulting in attenuation of ionic interactions^56^. Therefore, pressure is particularly sensitive to changes in hydration as the density of hydration water around charged residues is higher than that of bulk water^57^. Interestingly, conformations and configurations of DNA duplexes have been demonstrated to be relatively unaffected by pressure perturbation^58–61^, whereas an intramolecular, antiparallel DNA G-quadruplex structure has been reported to be more pressure-sensitive^62^. To study the pressure effect on the self-assembly of 5′-GMP, we first applied pressure-dependent FTIR measurements at various temperatures (Fig. 3). Upon pressurization, a blue shift of the IR bands is observable and can be explained by the pressure-induced compression of the bond lengths (Figs 3a and S3)^63^. Due to this intrinsic elastic pressure effect on the vibrational frequency, we decided to consider the IR band at ~1537 cm^−1^ for further analysis and plotted the absorbance intensity of the band maximum as a function of pressure (Fig. 3b). Again, we obtained an intensity decrease suggesting pressure-induced dissociation of the hydrogen-bonded species. However, the pressure-induced disassembly is less pronounced compared to the thermal-induced dissociation. At 278 K, dissociation could not be completed upon pressurization up to 1200 MPa. When the thermal disassembly is nearly complete at 308 K, no further pressure-induced assembly or disassembly of G-quartets is observable. Complementary high pressure ^1^H NMR measurements confirm that pressure populates the monomeric state (Figs 4 and S4). Upon pressurization up to 240 MPa, the tetrameric fraction is reduced from 56% to 26%, whereas the dimeric fraction diminishes from 22% to 11%, indicating that the G-quartets and the rather thermally unstable dimers feature similar pressure stabilities (Fig. 4b). The standard-state free energy change for the disassembly process of 5′-GMP, ${\rm{\Delta }}{G}_{{\rm{D}}}^{{\rm{0}}}$, was calculated from the mole fractions of monomeric and hydrogen-bonded species and plotted as a function of pressure (Fig. 4c). The pressure-dependence of ${\rm{\Delta }}{G}_{{\rm{D}}}^{{\rm{0}}}$ describes the standard molar volume change for the disassembly process of 5′-GMP, ${\rm{\Delta }}{V}_{{\rm{D}}}^{{\rm{0}}}$. We obtained ${\rm{\Delta }}{V}_{{\rm{D}}}^{{\rm{0}}}$ = −18 mL mol^−1^ at 278 K. The corresponding transition pressure value, *p*_m_, is 164 MPa (at ${\rm{\Delta }}{G}_{{\rm{D}}}^{0}({p}_{{\rm{m}}})$ = 0). ${\rm{\Delta }}{V}_{{\rm{D}}}^{{\rm{0}}}$ increases, whereas *p*_m_ decreases with elevated temperature (Suppl. Fig. S4). To address the question whether the length of the stacks and thus the stacking interaction is affected by pressure, we performed pressure-dependent small-angle X-ray scattering (SAXS) and diffusion-ordered NMR spectroscopy (DOSY). Owing to the high polydispersity within the system, as stacks of monomers, dimers and G-quartets are present in equilibrium, the SAXS data are interpreted in a qualitative manner, only. The overall high intensities of the scattering profile indicate the existence of large species (Fig. 5a). Upon pressurization, a slight decrease of the scattering intensity can be observed at 280 K, whereas the scattering profile at 308 K is marginally changed with pressure, suggesting that the longitudinal dimension of the monomer stacks is hardly affected by pressure. Complementary ^1^H DOSY NMR experiments yielded the molecular translational diffusion coefficients, *D*_t_, of stacks formed by 5′-GMP monomers and G-quartets as a function of pressure (Fig. 5b). The *D*_t_ values reveal that the stacking interaction and thus the longitudinal dimension of the G-quartet stacks are pressure-resistant up to about 240 MPa, independent of temperature. In contrast, at low temperature the number of stacks for 5′-GMP monomers is even increased with pressure, indicating dense packing of such assemblies.

Overall, our results show that pressure increases base stacking and induces lateral dissociation of dimer and G-quartet stacks of 5′-GMP into monomer stacks (Fig. 5c). In general, high pressure studies on DNA duplexes have revealed that pressure decreases the extent of charge neutralization by cations, reduces the Watson-Crick hydrogen-bond distance and favours base stacking, but leads to a very small net effect on the stability and conformation^58, 59, 64^. The attenuation of electrostatic interactions is explained by greater hydration of the cations and the phosphate groups under high-pressure conditions (electrostrictive effect). Consequently, the electrostatic repulsion between adjacent, negatively charged phosphates is also decreased, favouring stacking of neighbouring bases^64, 65^. Stacking of aromatic compounds has been shown to be accompanied by a negative volume change and is thus favoured at elevated pressures^66^. In case of the 5′-GMP self-assembly, the observed pressure resistance of the longitudinal dimension and thus the stacking interaction can be explained by the same mechanism. In the liquid-crystalline phase, where the 5′-GMP quartet stacks are hexagonally arranged, the stacking distance has been shown to be decreased upon compression^67^. Further, an acidic shift of the water pH upon pressurization would lead to partial protonation of the doubly charged phosphate groups, thus reducing the electrostatic repulsion between adjacent guanine bases^68^. In contrast, the typical pressure-induced strengthening of hydrogen bonds does not lead to a stabilization of the G-quartet structure, but rather a dissociation at elevated pressures. This finding suggests a volume increase upon G-quartet formation, which is unfavourable under high pressure conditions. The distance between the diagonal O6 atoms in the G-quartet is approximately 5 Å and the eight inwardly pointing guanine O6 atoms of two adjacent G-quartets span a volume of 40 Å^3^ ^38^. The Na^+^ ion has a diameter of 1.9 Å and a volume of 3.6 Å^3^. As the central channel formed by G-quartet stacks is only occupied by dehydrated cations and very few water molecules (diameter *d* = 2.75 Å)^69^, it is reasonable to assume that a significant number of water-inaccessible voids is present in the G-quartet stacks. Those voids will disappear upon dissociation of the G-quartet structure. Further volume reduction can be obtained from rehydration of the Na^+^ ions and the nucleobases after release from the channel, as the density of the hydration water is higher compared to that of bulk water^57, 70, 71^. Elimination of void volume, though of much lesser extent, in the dimer stacks upon pressure-induced formation of monomer stacks explains their pressure sensitivity as well.

### Effect of alkali salt on the thermal and pressure stability of 5′-GMP self-assemblies

**Figure 6:**
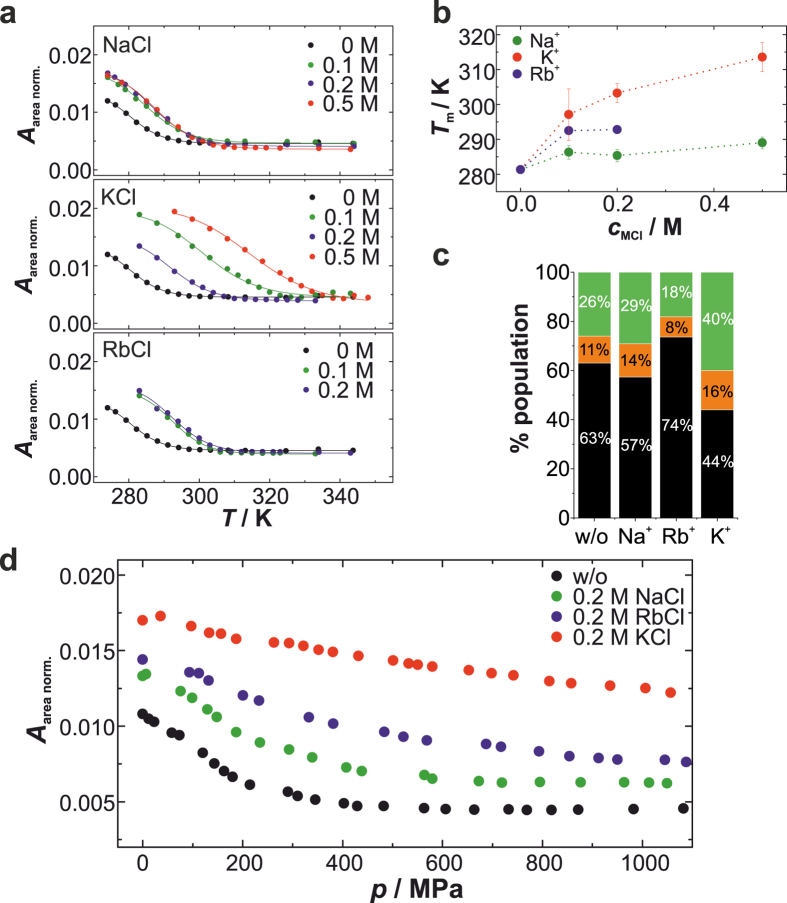
Effect of alkali salts on 5′-GMP self-assembly. (**a**) Area normalized absorbance intensities of the IR band at ~1537 cm^−1^ as a function of temperature. Various concentrations of additional alkali salts were added to 0.48 M Na_2_5′-GMP. (**b**) Melting temperature, *T*_m_, as a function of the alkali salt concentration. *n* = 2–3, error bars indicate mean ± s.d. The pointed lines are drawn to guide the eyes. (**c**) Population distribution of 0.48 M Na_2_5′-GMP in the presence of 0.2 M additional alkali salt at 278 K and 240 MPa obtained from ^1^H NMR. Green: G-quartet, orange: dimer, black: monomer. (**d**) Pressure-dependent absorbance intensities of the IR band at ~1537 cm^−1^ and at 288 K upon area normalization.

In a very elegant approach, using NMR titration experiments Wong and Wu have shown that the channel Na^+^ ions bound to the G-quartet stacks are replaced by K^+^ and Rb^+^ ions in a competitive equilibrium, whereas the Na^+^ ions bound to the peripheral phosphates remain unaffected^38^. This allows us to explore the thermal and pressure stability of 5′-GMP quartet stacks when the channel Na^+^ ions are gradually replaced by K^+^ and Rb^+^, respectively. Hence, we added increasing amounts of additional MCl to 0.48 M Na_2_5′-GMP. First, we performed temperature-dependent FTIR experiments and studied the thermal stability of the hydrogen-bonded 5′-GMP self-assemblies in the presence of the alkali salts, MCl = NaCl, KCl and RbCl (Suppl. Fig. S5). The IR bands assigned for guanine are not affected by the alkali salts, indicating that additional structures are not formed. The “melting” curves reveal that all three alkali salts are able to increase the thermal stability of the hydrogen-bonded 5′-GMP self-assemblies (Fig. 6a). While the melting temperature, *T*_m_, in the presence of additional NaCl is only slightly increased, additional KCl causes a concentration-dependent stabilization of G-quartets against temperature, indicating gradual replacement of the channel Na^+^ by K^+^ (Fig. 6b). In contrast, the addition of RbCl induces a moderate stabilization, which seems to be concentration-independent. RbCl could be tested only up to 0.2 M due to precipitation of 5′-GMP at higher concentrations of RbCl. The stabilization effect of these alkali cations follows the affinity order K^+^ > Rb^+^ > Na^+^ found for the channel site^38^, confirming the hypothesis that the channel cation determines the thermal stability of the G-quartet structure^38^. To examine the effect of the alkali salts on the pressure stability of the G-quartets, we performed pressure-dependent FTIR and ^1^H NMR experiments in the presence of 0.2 M additional MCl (Figs 6 and S6). We found KCl to be most effective against pressure-induced dissociation of G-quartets. At 240 MPa and 278 K, the population distribution is 40% G-quartets, 16% dimers, and 44% monomers (Fig. 6c). This is consistent with our complementary FTIR results revealing a stabilization trend of K^+^ > Rb^+^ > Na^+^ (Fig. 6d). This stabilization sequence against pressure can be explained by the concept of optimal packing. The K^+^ ion has a diameter of 2.66 Å and occupies consequently a larger volume of the channel cavity leading to reduced voids. Furthermore, owing to its low charge density and slightly hydrophobic nature^72^, K^+^ is very likely less densely hydrated and thus the volume reduction upon rehydration would be less compared to Na^+^. Both contributions result in a reduced $\Delta {V}_{{\rm{D}}}^{{\rm{0}}}$ and an improved pressure resistance. In the case of Rb^+^, the same argumentation should be valid, causing even more increased pressure stability. However, it is very likely that the channel Na^+^ ions are not completely replaced by Rb^+^, as the constant for the competition equilibrium has been found to be 1.8^38^. Thus, the observed stabilizing effect is achieved by both cations, Na^+^ and Rb^+^, in the channel cavity.

## Conclusions

In summary, we have presented to our knowledge the first comprehensive thermodynamic study on the molecular self-assembly of 5′-GMP. Using a combination of scattering and spectroscopic methods, we have studied the stability of supramolecular self-assemblies of 5′-GMP and the stabilization effect of alkali salts over a wide range of temperatures and pressures. We found that under abyssal conditions, where temperatures as low as 5 °C and pressures up to 1 kbar are reached, the G-quartet structure of Na_2_5′-GMP is stable. Beyond those conditions, the G-quartet stacks dissociate laterally into monomer stacks without changing the longitudinal dimension. Hydration, stacking interactions and packing defects play important roles in determining the stability of such supramolecular structures. In the presence of the alkali cations K^+^ and Rb^+^, which bind and replace the Na^+^ cation from the channel cavity of the G-quartet stacks, the temperature and pressure stability of the 5′-GMP quartets is significantly increased. As the G-quartets serve as building blocks of G-quadruplexes, our results provide also insights into how different forces determining the folding of G-quadruplexes are modulated by temperature and pressure. Especially, the pressure effect on the G-quartets can be extrapolated to their oligomeric analogues and help to explain the pressure sensitivity of G-quadruplexes in general^62, 71^.

## Methods

### Sample preparation

Disodium guanosine 5′-monophosphate salt hydrate (Na_2_5′-GMP, >99% purity) from yeast was purchased from Sigma-Aldrich. Stock solutions of GMP (35 wt%) and the alkali salts (1 M) were prepared in doubly distilled water or D_2_O and filtered (0.2 µm PVDF membrane). 5′-GMP samples were dissolved overnight to ensure homogeneity. The concentration of the stock solution was determined from UV-absorption measurements at a wavelength of 260 nm (20,000-fold dilution, *ε*_260_ = 10674 [M cm^−1^) and adjusted to the appropriate concentration^31^. For FTIR measurements, stock solutions of Na_2_5′-GMP in D_2_O are lyophilized twice before filtration in order to remove H_2_O from the crystal water. For NMR measurements, Na_2_5′-GMP was dissolved in 1:1 H_2_O:D_2_O, the solution was lyophilized, filtered with 0.2 μm spin filters and re-dissolved in 1:1 H_2_O:D_2_O. The Na_2_5′-GMP concentration was 0.48 M in all NMR experiments.

### Dynamic light scattering

Temperature-dependent DLS experiments were performed using a Malvern Zetasizer Nano S. Scattered light of a 632.8 nm helium-neon laser was analysed at an angle of 173°. The sample was equilibrated for 5 min at each temperature. A total of 3 scans were collected for the sample at each temperature. All samples were analysed in triplicate. For the polydisperse samples, the method of cumulants and a symmetric spherical model were used. The translational diffusion coefficient, *D*_t_, values were obtained from the acquired correlograms using the software Zetasizer (Malvern). The *D*_t_ values were further used to calculate the apparent hydrodynamic diameter, *d*_app,h_, considering the Stokes-Einstein equation. The temperature dependence of the dispersant viscosity (H_2_O) was taken into consideration. We used the following relationship log *η*(H_2_O) = 8.128–0.0447 *T* + 0.0000579*T* ^2^, where the unit for *η* is centipoise (cP, 1 cP = 0.001 kg m^−1^ s^−1^).

### Temperature- and pressure-dependent FTIR spectroscopy

Temperature-dependent FTIR measurements were performed using a Nicolet 5700 (Thermo Fisher Scientific, USA) equipped with a liquid-nitrogen-cooled MCT detector (HgCdTe) in the wavenumber range of 4000–1100 cm^−1^ and in the temperature range of 1–70 °C. The sample was placed between two CaF_2_ windows separated by a mylar spacer (thickness, 50 µm) and assembled in a temperature cell which is connected to an external water bath. The temperature was measured with a digital thermometer placed in the sample cell (accuracy: ±0.5 °C). The sample was equilibrated for 5 min at each temperature before spectra were collected. Pressure-dependent FTIR spectra were collected using a Nicolet 6700 (Thermo Fisher Scientific, USA) equipped with a liquid-nitrogen-cooled MCT detector (HgCdTe) in the wavenumber range of 4000–650 cm^−1^. Pressure up to 1200 MPa was applied using a P-series diamond anvil cell (DAC) with type IIa diamonds (High Pressure Diamond Optics Inc., Tucson, USA). A 20 µm thick gasket of CrNi steel with a 0.3 mm drilling was placed between the two diamonds holding 2.5 µL of the sample. The temperature of the DAC was controlled by an external water bath and the internal temperature was measured with a digital thermometer. BaSO_4_ was used as an internal pressure gauge. The stretching vibration of SO_4_^2−^ (~983 cm^−1^) is pressure-dependent^73^. For each temperature or pressure, 128 scans were collected with a spectral resolution of 2 cm^−1^ and averaged. Data analysis was carried out using the GRAMS software (Thermo Electron). After buffer subtraction and baseline correction in the range of 1710–1630 cm^−1^ (band for the C=O stretch vibration) and 1610–1518 cm^−1^ (band for the C=N ring vibration), those bands were area normalized and smoothed. All measurements were performed at least in duplicate.

Assuming an apparent thermodynamic equilibrium between hydrogen-bonded and monomer assemblies, a Boltzmann function was fitted to the temperature-dependent band intensities at 1673, 1592, 1568 and 1537 cm^−1^ using

1 $$I(T)=\frac{{I}_{{\rm{1}}}-{I}_{2}}{1+{{\rm{e}}}^{-(\frac{1}{{T}_{{\rm{m}}}}-\frac{1}{T})\cdot \frac{{\rm{\Delta }}{H}_{{\rm{vH}}}^{0}}{R}}}+{I}_{2}$$

where *I*_1_ and *I*_2_ are the plateau values of the IR band intensities of the monomeric and hydrogen-bonded state, respectively. *T*_m_ is the melting temperature and ${\rm{\Delta }}{H}_{{\rm{vH}}}^{{\rm{0}}}$ the standard van’t Hoff enthalpy for the dissociation reaction.

### High pressure Synchrotron SAXS

Pressure-dependent SAXS measurements were performed at the ESRF beamline ID 02 (Grenoble, France) in a home-built high pressure cell with diamond windows^74^. The sample volume was 10 µl. The energy used was 16 keV and the sample to detector distance was 2.4 m. The samples were exposed to the beam for 0.25 s for each measurement. No radiation damages were detected within the total exposure time of a complete pressure series. Data were collected in 50 MPa steps. The scattering data were background corrected and analysed using the SAXSutilities software package provided by ESRF^75^.

### High pressure NMR and DOSY NMR

All NMR spectra were acquired on a Bruker Avance III 600 MHz spectrometer with a standard 5 mm O.D. ceramic tube from Daedelus Innovations. Hydrostatic pressure was applied to the sample using the Xtreme Syringe Pump (Daedelus Innovations). A commercial ceramic zirconia high-pressure NMR cell and an automatic pump system (Daedalus Innovations, Philadelphia, PA) were used to vary the pressure in the 0.1 to 240 MPa range^76^. Longitudinal relaxation times *T*_1_ were measured for the H8 protons using an inversion recovery experiment, and the data were analysed using the relaxation module of Bruker’s Topspin software. The *T*_1_ values at various temperatures and pressures are given in Supplementary Table S1. The range of *T*_1_ values varied from 1.9 s at 35 °C to 0.8 s at 5 °C for the tetramer, and from 1.4 s at 35 °C to 1 s at 5 °C for the monomer. Conversely, pressure had a very little effect on *T*_1_. Standard ^1^H NMR experiments were performed with the zgesgp pulse sequence using excitation sculpting for solvent suppression. At each pressure, 16 scans were collected with a recycle delay of 10 s at 15, 25 and 35 °C and 32 scans were collected with a recycle delay of 5 s at 5 °C. The data were processed and analysed on Bruker Topspin software version 3.5 pl 5. For ^1^H diffusion experiments, the stebpgp1s pulse sequence was used. The pulsed field gradient duration (δ) was 10 ms, the diffusion period (Δ) was 250 ms and the variable gradient strength (G) was 34.05 T/cm. For the gradient, 32 scans were collected for each of 32 linear incremental steps from 2% to 95% of the gradient strength, with a recycle delay of 5 s. The data were analysed using the ‘eddosy’ tool on Bruker Topspin software version 3.5 pl 5. The temperature and pressure dependence of water’s dynamic viscosity was considered^77^.

The standard-state free energy of dissociation, ${\rm{\Delta }}{G}_{{\rm{D}}}^{{\rm{0}}}$, was determined by

2 $${\rm{\Delta }}{G}_{{\rm{D}}}^{{\rm{0}}}(p)=-RT\,\mathrm{ln}\,{K}_{{\rm{app}}}=-RT\,\mathrm{ln}\,\frac{{f}_{{\rm{m}}}}{{f}_{{\rm{hb}}}}$$

where the apparent equilibrium constant, *K*_app_, for the disassembly reaction is given by the ratio of the mole fractions of monomer and hydrogen-bonded 5′-GMP assemblies (dimers and G-quartets), *f*_m_ and *f*_hb_.

The standard-state molar volume change of the disassembly process of 5′-GMP, ${\rm{\Delta }}{V}_{{\rm{D}}}^{{\rm{0}}}$, was calculated from

3 $$(\frac{\partial {\rm{\Delta }}{G}_{{\rm{D}}}^{{\rm{0}}}}{\partial p})={\rm{\Delta }}{V}_{{\rm{D}}}^{{\rm{0}}}$$

that is the slope of the linear plot of ${\rm{\Delta }}{G}_{{\rm{D}}}^{{\rm{0}}}(p)$.

### Data availability

The data that support the findings of this study are available from the corresponding author on reasonable request.

## Electronic supplementary material

Supplementary Information
