# Supplementary material for: Temperature and pressure limits of guanosine monophosphate self-assemblies
Source: Sci Rep. 2017 Aug 29;7:9864. doi: 10.1038/s41598-017-10689-0 (PMC5574928; doi:10.1038/s41598-017-10689-0)
Supplement: Supplementary file 1 — Supplementary Information [file 41598_2017_10689_MOESM1_ESM.pdf]

SUPPORTING INFORMATION

Temperature and pressure limits of guanosine monophosphate self-assemblies

Mimi Gao[1], Balasubramanian Harish[2], Melanie Berghaus[1], Rana Seymen[1], Loana Arns[1], Scott A. McCallum[3], Catherine A. Royer[2], Roland Winter[1]*

[1]Physical Chemistry I - Biophysical Chemistry, Faculty of Chemistry and Chemical Biology, Technical University Dortmund, Otto-Hahn-Street 4a, 44227 Dortmund, Germany

[2]Center for Biotechnology & Interdisciplinary Studies, Rensselaer Polytechnic Institute, Troy, New York 12180, United States

[3]NMR Facility Center for Biotechnology and Interdisciplinary Science, Rensselaer Polytechnic Institute, Troy, New York, United States

ADDITIONAL FIGURES

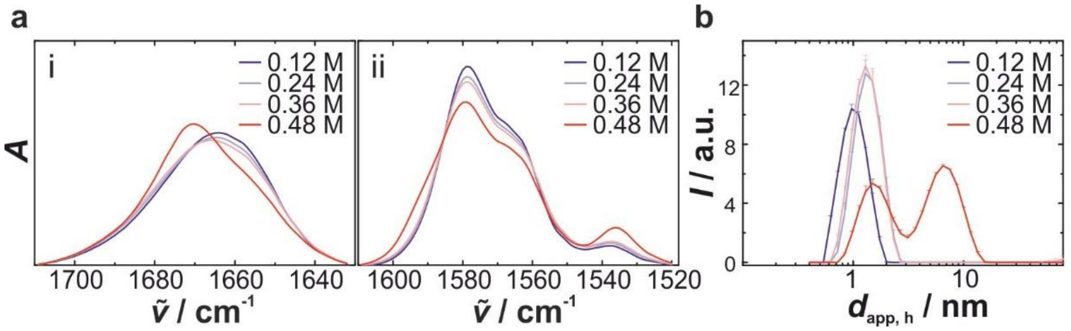

Figure S1: Concentration dependence of disodium 5'-guanosine monophosphate ($Na_2$5'-GMP) self-assembly. (a) Area normalized FTIR spectra of $Na_2$5'-GMP in D_2O at different concentrations: i) in the range of 1700-1640 cm^{-1} at 296 K (C=O stretch vibration), ii) in the range of 1600-1520 cm^{-1} at 296 K (C=N ring vibration). (b) DLS diagrams of $Na_2$5'-GMP in H_2O at different concentrations and 296 K. Hydrodynamic diameters calculated based on spherical symmetric particles and using the method of cumulants. Error bars indicate mean ± s.d. of three scans.

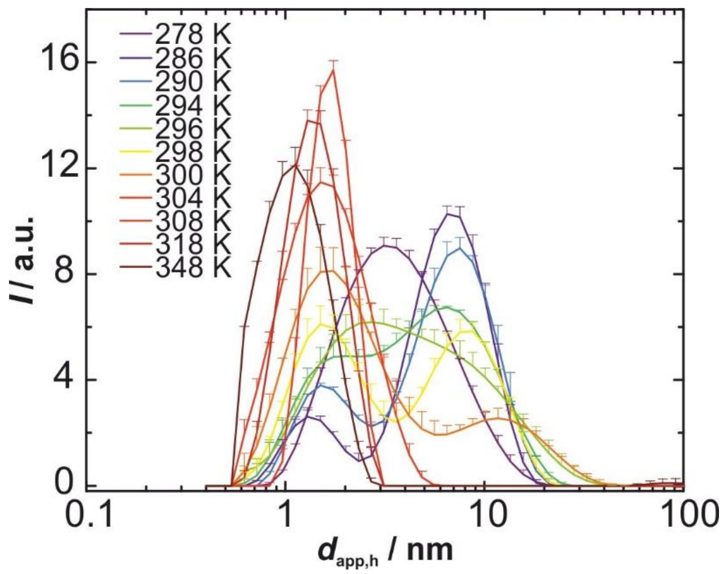

Figure S2: DLS diagrams of 0.48 M $Na_2$5'-GMP in H_2O as a function of temperature. Hydrodynamic radii calculated based on spherical symmetric particles and using the method of cumulants. Error bars indicate mean ± s.d. of three scans.

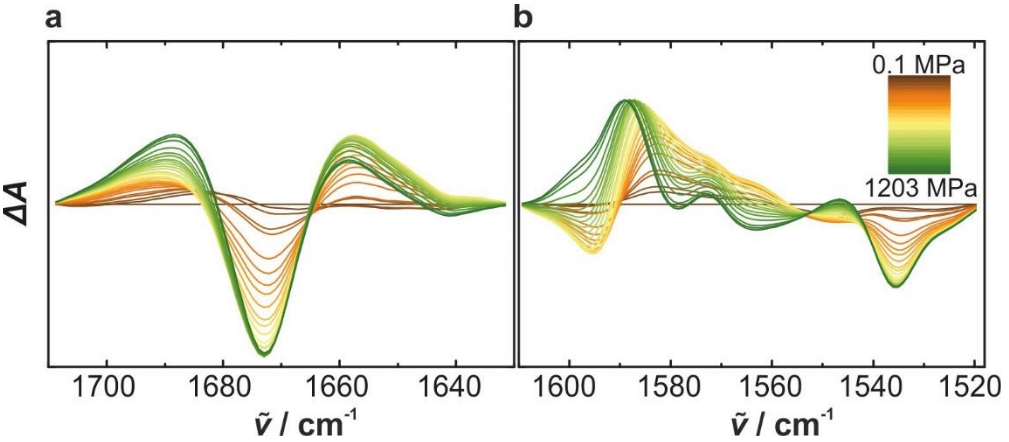

Figure S3: Pressure-dependent difference FTIR spectra of 0.48 M $Na_2$5'-GMP in D_2O and at 288 K for a) the C=O stretch vibration, and b) the ring vibrations (C=C, C=N). The absorption spectrum at 0.1 MPa was subtracted for the spectra at high pressure values.

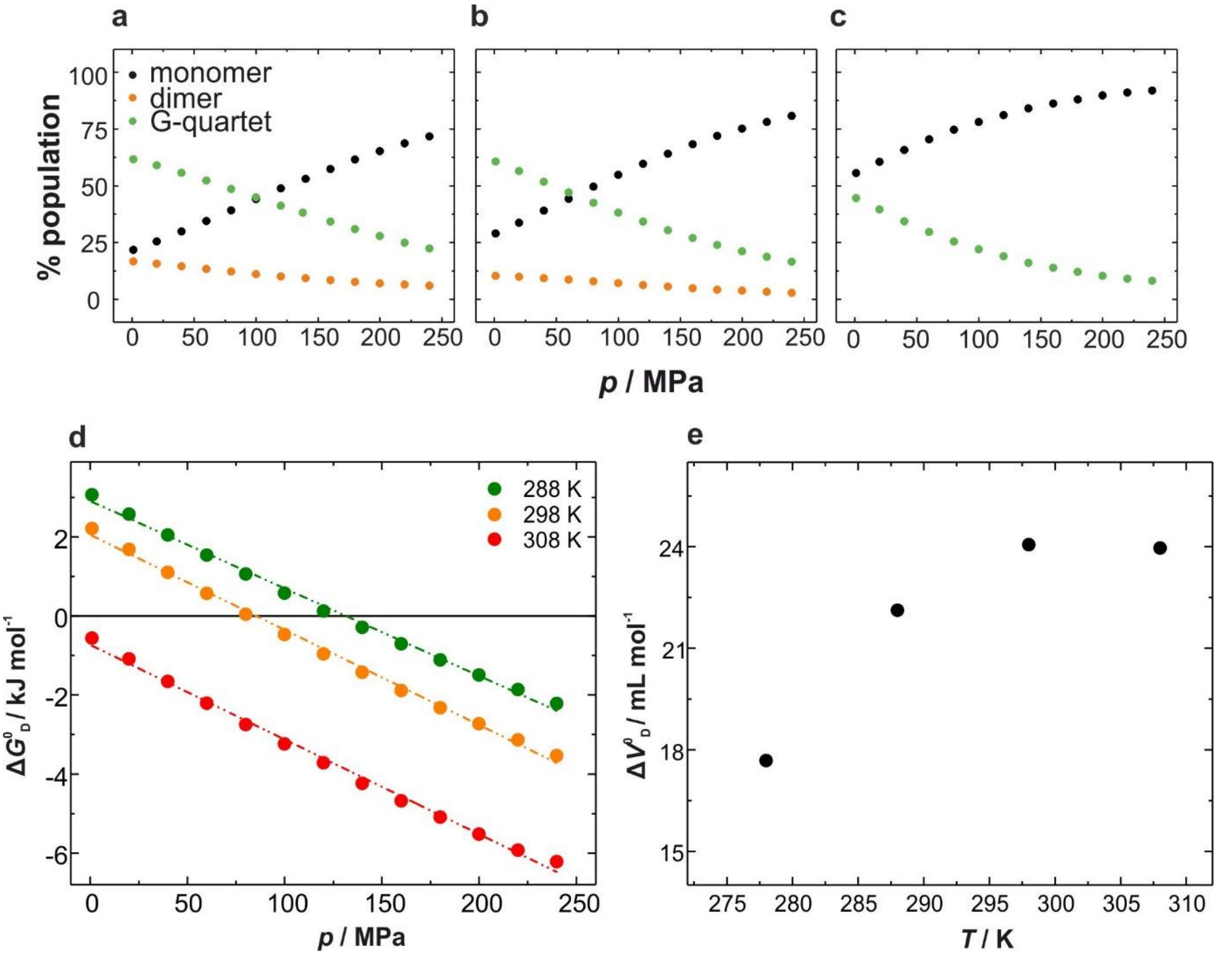

Figure S4: Pressure-dependent population distribution of $Na_2$5'-GMP as a function of temperature obtained from ^{1}H NMR measurements: (a) at 288 K, (b) 298 K, and (c) 308 K. (d) Pressure-dependent standard free energy of the 5'-GMP disassembly at various temperatures. The dashed lines are the least-square fit to the data and describe the standard molar volume changes of the dissociation process as shown in (e).

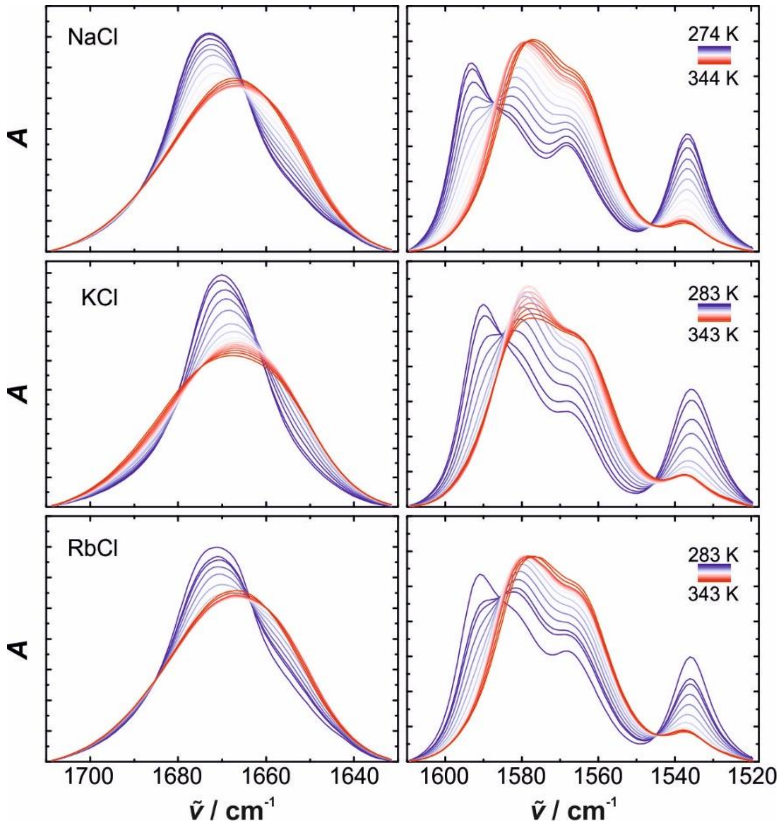

Figure S5: Temperature-dependent area normalized FTIR spectra of 0.48 M $Na_2$5'-GMP in D_2O and in the presence of 0.2 M additional alkali salt. The spectra were taken at 0.1 MPa. *Left* C=O stretch vibration, *right* ring vibrations.

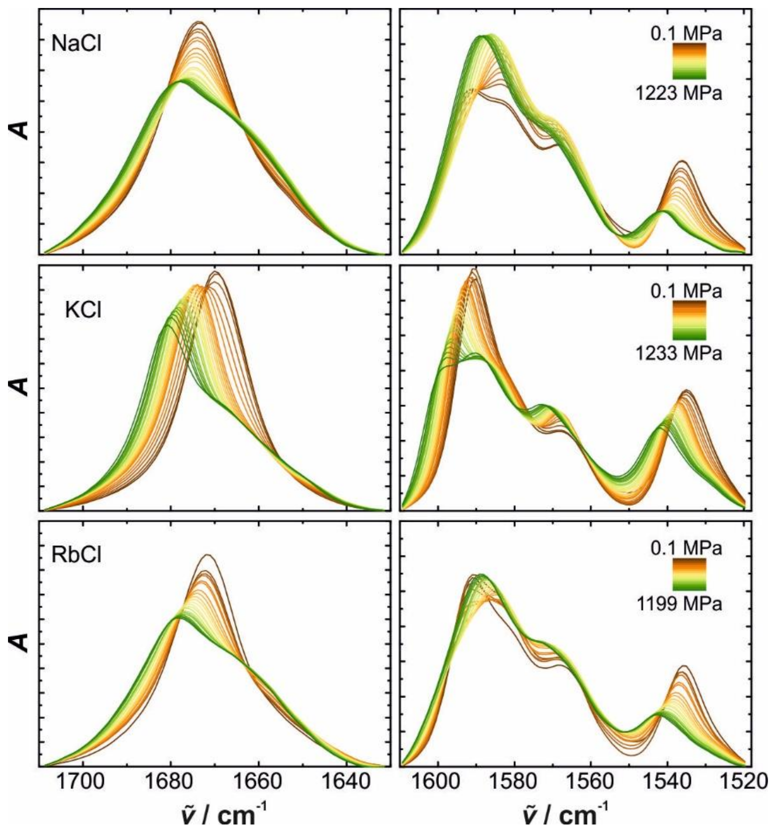

Figure S6: Pressure-dependent area normalized FTIR spectra of 0.48 M $Na_2$5'-GMP in D_2O and in the presence of 0.2 M additional alkali salt. The spectra were taken at 288 K. *Left* C=O stretch vibration, *right* ring vibrations.

ADDITIONAL TABLES

Table S1: [1]H longitudinal relaxation time, T_1 (in s), for 0.48 M $Na_2$5'-GMP measured using an inversion recovery experiment.

pressure (MPa)	monomer	dimer	(S)-quartet	(N)-quartet
T = 308 K				
1	1.43	-	1.91	1.86
120	1.35	-	1.90	1.88
240	1.37	-	1.81	1.80
T = 298 K				
1	1.43	1.43	1.79	1.79
120	1.39	1.38	1.78	1.79
240	1.36	1.35	1.68	1.70
T = 288 K				
1	1.31	1.31	1.44	1.48
120	1.33	1.32	1.47	1.51
240	1.31	1.29	1.43	1.47
T = 278 K				
1	0.95	0.93	0.81	0.88
120	1.00	0.95	0.87	0.93
240	1.04	0.93	0.82	0.88
